# Infrared Spectroscopy in Gynecological Oncology: A Comprehensive Review of Diagnostic Potentials and Challenges

**DOI:** 10.3390/ijms25115996

**Published:** 2024-05-30

**Authors:** Charlotte Delrue, Sander De Bruyne, Matthijs Oyaert, Joris R. Delanghe, Rafael Noal Moresco, Reinhart Speeckaert, Marijn M. Speeckaert

**Affiliations:** 1 Department of Nephrology, Ghent University Hospital, 9000 Ghent, Belgium; charlotte.delrue@ugent.be; 2 Department of Clinical Biology, Ghent University Hospital, 9000 Ghent, Belgium; sander.debruyne@uzgent.be (S.D.B.); matthijs.oyaert@uzgent.be (M.O.); 3 Department of Diagnostic Sciences, Ghent University Hospital, C. Heymanslaan 10, 9000 Ghent, Belgium; joris.delanghe@ugent.be; 4 Graduate Program in Pharmaceutical Sciences, Center of Health Sciences, Federal University of Santa Maria, Santa Maria 72500-000, Brazil; rnmoresco@ufsm.br; 5 Department of Dermatology, Ghent University Hospital, 9000 Ghent, Belgium; reinhart.speeckaert@uzgent.be; 6 Research Foundation-Flanders (FWO), 1000 Brussels, Belgium

**Keywords:** infrared spectroscopy, gynecological tumors, near-infrared, mid-infrared

## Abstract

The early detection of gynecological cancers, which is critical for improving patient survival rates, is challenging because of the vague early symptoms and the diagnostic limitations of current approaches. This comprehensive review delves into the game-changing potential of infrared (IR) spectroscopy, a noninvasive technology used to transform the landscape of cancer diagnosis in gynecology. By collecting the distinctive vibrational frequencies of chemical bonds inside tissue samples, Fourier-transform infrared (FTIR) spectroscopy provides a ‘molecular fingerprint’ that outperforms existing diagnostic approaches. We highlight significant advances in this field, particularly the identification of discrete biomarker bands in the mid- and near-IR spectra. Proteins, lipids, carbohydrates, and nucleic acids exhibited different absorption patterns. These spectral signatures not only serve to distinguish between malignant and benign diseases, but also provide additional information regarding the cellular changes associated with cancer. To underscore the practical consequences of these findings, we examined studies in which IR spectroscopy demonstrated exceptional diagnostic accuracy. This review supports the use of IR spectroscopy in normal clinical practice, emphasizing its capacity to detect and comprehend the intricate molecular underpinnings of gynecological cancers.

## 1. Introduction

Cervical, endometrial, ovarian, and breast cancers are gynecological malignancies that pose a large global health burden, with varying epidemiological patterns reflecting population heterogeneity in lifestyle, environmental, and genetic variables. The generic character of early symptoms makes early diagnosis of many tumors difficult, resulting in late-stage detection and lower survival rates. Conventional diagnostic techniques such as imaging and biopsy are proficient, yet they have restrictions regarding their sensitivity and specificity, and they require invasive procedures. Moreover, traditional anatomical imaging techniques such as ultrasound (US), computed tomography (CT), and magnetic resonance imaging (MRI) are at the forefront of guiding surgical procedures. However, tiny metastases or differentiation between malignant tumors can be missed by these techniques because of their shortcomings in sensitivity, resolution, and contrast production.

Tumor diagnosis and its treatment could be improved by “molecular imaging” by its ability to view biological activity at the cellular and molecular levels. Infrared (IR) spectroscopy examines the molecular composition of cells and tissues, which leads to a thorough understanding of biological processes occurring both at the molecular and cellular levels. Some molecules will absorb IR light at particular wavelengths, resulting in a distinct spectral pattern that serves as a “molecular fingerprint”. The capacity of IR spectroscopy to identify minute biochemical changes prior to their manifestation as anatomical alterations in tissues permits the prompt detection of cancer, hence augmenting the likelihood of efficacious therapy and favorable consequences for patients. By charting the biochemical landscape of illness progression, this non-invasive, label-free method provides a key window for intervention in addition to aiding in the early detection of pathological situations. The optical molecular imaging technique in the therapeutic setting is primarily comprised of a surgical navigation system and an appropriate tracer. The near-infrared (NIR) light wavelengths, which fall between 650 and 900 nm, are selected because they have a tissue optical window that allows light to enter cells deeply while absorbing very little of it [1]. Besides NIR-spectroscopy, covalently bound molecules vibrate at specific frequencies with enhanced amplitude when a bio-sample receives mid-infrared (MIR) radiation (4000–200 cm^−1^). The wavelength at which absorption takes place indicates the formation of the chemical bonds of these molecules, while the intensity of the absorption is correlated with their concentration [2]. Within the bio-fingerprint region of the MIR spectrum, the fundamental frequencies of key biomolecules are present. Proteins predominantly account for absorption bands between 1650 cm^−1^ and 1665 cm^−1^, at 1550 cm^−1^, and between 1310 cm^−1^ and 1200 cm^−1^, which correspond to amide I (C=O stretching), amide II (N–H bending, C–H stretching, C–O bending, and C–C and N–C stretching), and amide III (C–H/N–H deformation), respectively. Lipids are represented by bands between 1467 cm^−1^ and 1400 cm^−1^ (C–H scissoring or CH_2_ and CH_3_ and C=O stretching of –COO−), and at approximately 1070 cm^−1^ (C–O–C, CO–O–C symmetric stretching). Carbohydrate-related bands appear at 1173 cm^−1^, 1154 cm^−1^ (symmetric stretching of C–O, coupled with C–O–H bending), 1041 cm^−1^ and 1055 cm^−1^ (symmetric C–O–C stretching), and 1023 cm^−1^ (symmetric C–O stretching). Bands associated with nucleic acids, phospholipids, and nucleotides are evident at approximately 1250 cm^−1^ to 1220 cm^−1^ (asymmetric PO_2_^−^ stretching), around 1085 cm^−1^ (symmetric PO_2_ and symmetric CO–O–C stretching), and 900 cm^−1^ to 800 cm^−1^ (C=C, C=N, and C–H vibrations in ring structures) [3,4,5]. When using MIR spectroscopy in vivo, the presence of water in tissues remains a significant challenge. In hydrated tissues, the strong H–O–H bending vibration at 1610 cm^−1^ tends to dominate the bio-fingerprint region, overshadowing critical information from the amide I (1601 cm^−1^), amide II (1645 cm^−1^), and other nearby bands. It is beneficial to concentrate on a different part of the MIR spectrum to overcome this limitation when analyzing in vivo MIR spectral data [5].

In this review, we will concentrate on the significant contributions of studies that explore the role of IR spectroscopy in diagnosing gynecological tumors through the analysis of biofluid samples, deliberately excluding studies focused on IR spectroscopy-based imaging techniques.

## 2. Methods

We looked for pertinent papers published between 1990 and 2024 in the PubMed Database for this narrative review. “IR spectroscopy”, “diagnosis”, and “gynecological oncology” were the search terms. To find additional studies, we also examined the reference lists of relevant papers. The following criteria were used to determine which publications were included: (1) original research articles published in peer-reviewed journals; (2) studies examining the connection between gynecological malignancies and IR spectroscopy; and (3) studies summarizing the technical aspects of IR spectroscopy. Research written in languages other than English, unpublished studies, event abstracts, and journals lacking an English abstract were considered for exclusion. We collected data from the included studies, paying particular attention to study design, sample size, patient characteristics, and results.

## 3. Cervical Cancer

Cervical cancer is primarily caused by persistent infection with high-risk human papillomaviruses (HPV). It is ranked as a significant public health issue globally, especially in low- and middle-income countries where screening and vaccination programs are less accessible. Annually, more than 400,000 new cases of invasive cervical cancer are identified globally, accounting for almost 10% of all cancer cases in women. A decrease in incidence in high-income countries has been established by Papanicolaou (Pap) smears and HPV testing. However, despite the availability of effective screening methods, challenges in early diagnosis persist. Cervical biopsy and analysis of the stained tissue are used to make a conclusive diagnosis. The diagnostic accuracy of a biopsy exceeds that of the Pap test, as it maintains the anatomical structure of the tissue, enabling the assessment of pathological characteristics in connection with histological architecture [6].

The amide I mode, which is visible at about 1650 cm^−1^ and changes with the secondary structure of the protein, is one of the hallmarks of protein structures that dominate the FTIR spectrum of an ectocervical cell. This specific mode includes a complicated set of vibrations that include the NH_2_ group from the in-plane peptide bond bending and the carbonyl (C=O) group stretching. This band appears at approximately 1665 cm^−1^ for random coils and β-turns, 1650 to 1655 cm^−1^ for α-helical forms, and roughly 1635 cm^−1^ for β-pleated sheets, depending on the secondary structure of the protein (Table 1) [6].

Proteins are also responsible for the second most prominent signature, the amide II mode, which appears at roughly 1544 cm^−1^. The primary mechanisms involved in this mode are C–N stretching from the peptide link and NH_2_ in-plane bending. The stretching of carboxylate at 1400 cm^−1^ and the bending modes of methyl/methylene groups at 1450 cm^−1^ are further protein-related characteristics found in the FTIR spectrum. The amide III mode is detected at about 1305 cm^−1^ and is mainly caused by NH_2_ in-plane bending vibrations, together with CH_2_ deformations of aliphatic amino acids [7]. The FTIR spectrum of ectocervical cells is significantly influenced by their high glycogen content, with prominent spectral features of this molecule visible around 1055, 1080, and 1150 cm^−1^. These peaks are attributed to the stretching vibrations of C–O groups within the glycogen structure. Lipids contribute to a distinctive peak at 1735 cm^−1^ in the spectrum, which is attributed to the stretching vibration of the ester carbonyl group. Lipid-related features within the 3000 to 2800 cm^−1^ range are due to CH stretching vibrations, attributed to the stretching modes of the methyl/methylene groups, with their bending vibrations observed near 1400 cm^−1^. For nucleic acids, key vibrations are seen at 1244 cm^−1^ and 1080 cm^−1^, corresponding to the asymmetric- and symmetric-stretching vibrations of the phosphodiester bonds in the backbone of nucleic acids. The place where the asymmetric phosphodiester stretch is located varies within the DNA structure. This band is present at 1244 cm^−1^ in the A-DNA form, which is generally found in dehydrated cells and tissues. In contrast, the band moves to 1225 cm^−1^ in the B-DNA form, which is indicative of living, hydrated cells [10].

With the publication of a groundbreaking study by Wong et al. in the early 1990s, the use of FTIR spectroscopy in the analysis of tissue slices and individual cells began to receive substantial attention [11]. In this work, cells obtained with an Ayre spatula and cytobrush were used to diagnose cervical cancer using FTIR spectroscopy. Normal tissue (*n* = 136), dysplastic tissue (*n* = 8), and carcinoma in situ (*n* = 12) revealed distinct spectral variations in the phosphodiester and glycogen regions (1300–950 cm^−1^). The IR spectra of cells from normal individuals were consistent across samples but showed distinct differences when compared to those from individuals with cancer or dysplasia. Specifically, in the cancerous samples, significant alterations in the intensity of the glycogen-related spectral bands at 1025 cm^−1^ and 1047 cm^−1^, along with the bands at 1082 cm^−1^, 1244 cm^−1^, the C–O stretching vibration at 1155 cm^−1^, and the spectral feature at 1303 cm^−1^ were observed. In addition, there were considerable shifts in the peaks that typically occur at 1082 cm^−1^, 1155 cm^−1^, and 1244 cm^−1^, as well as the emergence of a new band at 970 cm^−1^. Further examination of these bands revealed profound modifications in the biochemistry of cervical cancer tissues, including changes in hydrogen bonding within phosphodiester groups of nucleic acids and C–OH groups of proteins, as well as in the structural organization of lipid methylene chains. The IR spectra for dysplasia samples indicated similar biochemical alterations to those seen in cancerous samples, albeit to a lesser extent, with the exception that the phosphodiester-related peak at 1082 cm^−1^ remained unshifted. These spectral changes correlate with the histological transformation from normal cervical epithelium through dysplasia to cancer [11].

A study encompassing nearly 750,000 FTIR spectra obtained from cervical biopsy samples showed that the FTIR method could identify dysplastic cells classified by pathology as cervical intraepithelial neoplasia grade 1 (CIN I). Through examining various spectral ranges and juxtaposing the findings with histological data, the amide I and II region (1740–1470 cm^−1^) was identified as crucial for linking anatomical and histopathological characteristics of tissue with spectral groupings [6]. FTIR microscopy was used to distinguish HPV-infected cells from non-infected ones by looking for changes in the amide I and II modes. This implies that the virus has caused a consistent structural alteration in the cell. Furthermore, protein synthesis may function as a pre-emptive marker for dysplasia, which could elucidate the reason behind the striking distinction between malignant and healthy tissue when employing solely the amide I and II modes in cluster analysis [12]. Using principal component analysis (PCA) for spectral analysis of cervical specimens from 396 females followed by chi-square testing, statistically significant correlations were found between the FTIR spectroscopy-predicted malignancy and the clinically diagnosed malignancy (*p* < 0.0001), as well as conditions labeled as “atrophy” (*p* < 0.001), “atypical with bloody smear” (*p* < 0.05), “atypical with atrophic pattern” (*p* < 0.05), and “dysplasia” (*p* < 0.05). According to these results, the diagnostic efficiency of FTIR spectroscopy for cervical cancer yielded a sensitivity of 79%, a specificity of 77%, a positive predictive value of 15%, and a negative predictive value of 98.6% [8]. In another study, FTIR spectroscopy was used to examine 35 cervical tissue samples, which comprised 17 cases of squamous cell carcinoma, 5 cases of adenocarcinoma, and 13 normal tissue samples. A total of 18 spectral bands were found to be present in all three tissue categories with a high frequency (>80%), indicating that these bands may act as unique IR spectral markers for cervical tissues. Among various tissue types, there were statistically significant differences in the relative absorbance ratios, especially at wavelengths of 1080, 1238, 1314, 1339, 1397, 1454, 1541, 1647, 2854, 2873, 2926, and 2958 cm^−1^ (*p* < 0.05) [9]. A second study involving FTIR microspectroscopy and PCA on exfoliated cervical cells from 272 patients produced six spectra of each sample, which were then classified into two groups (type 1 and type 2), based on their spectral characteristics. Type 1 spectra showed patterns typical of normal epithelial cells, highlighted by strong glycogen bands at 1022 cm^−1^ and 1150 cm^−1^, along with a notable symmetric phosphate stretch at 1078 cm^−1^. In contrast, type 2 spectra were indicative of possible dysplastic or malignant changes. These spectra were characterized by evident symmetric and asymmetric phosphate modes and a decrease in glycogen band strength. Among the 272 patients, only type 1 spectra across all six recordings were observed in 68.6% of the samples, whereas 29.4% had at least one type 2 spectrum among their six spectra, and 2% produced inconclusive results. From those with exclusively type 1 spectra, 86% received a normal Pap smear diagnosis without the need for further biopsy, 7% were identified as abnormal through biopsy, 5% were confirmed normal by biopsy, and 2% remained undetermined. Of the samples that included at least one type 2 spectrum, 71% had previously shown abnormal Pap smear results. A biopsy followed for these cases, with 87% being confirmed as abnormal. The link between type 2 spectra and cell abnormalities was reinforced by the comparison with spectra from the HeLa cell line of malignant cultured cells, which resembled the type 2 spectra pattern in the 1300–950 cm^−1^ range. PCA analysis on a streamlined data set produced a score plot distinctly separating the two types of spectra visually identified [13]. Although relevant, it is difficult to directly compare the spectroscopic results to the current PAP smears or HPV tests. The HPV tests and Pap smears can detect cervical cancer with a sensitivity of 90–95% and 55–80%, respectively [14]. Current research on IR spectroscopy has demonstrated inconsistent sensitivity and specificity values, often attributed to limited sample sizes. Therefore, it is imperative to conduct comprehensive, well-designed studies on a larger scale, incorporating diverse populations and established methodologies.

The majority of the studies mentioned here primarily rely on the variations in absorbance of key nucleic acid vibrational modes to distinguish between normal and dysplasia-diagnosed samples, often applying a multivariate method for classification. Separate investigations have suggested that the spectral changes identified might not directly correlate with the quantity and molecular composition of dysplastic cells themselves, but rather with other elements, such as inflammation, the ratio of dividing-to-non-dividing cells [15,16], and the general divisional activity of the cells [17]. Owing to the similarity in spectral patterns between different cells, multivariate techniques have been used to reveal finer structures in the data. These methods make it easier to categorize spectra according to their variance, proving that they are capable of objective diagnosis devoid of subjective interpretation bias [13].

By detecting unique spectrum fluctuations in tissues, ATR-FTIR spectroscopy has demonstrated potential in the diagnosis of cervical cancer. Nevertheless, a careful evaluation of the studies identifies several shortcomings. A lot of studies have a relatively small sample size. For example, Wong et al. [11] looked at only 136 normal tissues, 12 cancer tissues, and 8 dysplastic tissues. Small sample sizes not only underestimate diagnostic accuracy, but they can also mask variability found in larger, more diverse populations. Selection biases are particularly clear because many studies focus on specific cohorts, limiting how broadly relevant the findings may be. The studies usually lack diversity in terms of disease stages and demography, which can skew the results. Furthermore, spectral results may be influenced by factors such as the ratio of dividing-to-non-dividing cells, inflammation, and HPV status. To ensure that the promised high sensitivity and specificity are accurate and reliable, these variables must be carefully monitored and controlled. The early findings will be corroborated by standardizing the approach and undertaking more comprehensive multicentric studies, hence improving the precision of ATR-FTIR spectroscopy as a cervical cancer detection technology.

## 4. Endometrial Cancer

Endometrial cancer is the most common gynecological malignancy in developed countries. Partly due to increasing life expectancy and obesity rates, it exhibits rising incidence rates. In 2020, the incidence of new endometrial cancer cases rose to over 417,300, and the number of deaths escalated to more than 97,000. Despite this, there is no widespread recommendation for endometrial cancer screening at present. The main reason for this is the significantly low specificity, which varies between 36% and 68%, and the low positive predictive values, ranging from 4% to 9.6% [18]. Following symptoms of postmenopausal bleeding, diagnosis primarily hinges on histopathological evaluation but early detection remains challenging, especially in asymptomatic stages or in women with atypical presentations. The variability in the biological behavior of the tumor and the invasive nature of current diagnostic procedures add further complexity. Estrogen-dependent (type I) and estrogen-independent (type II) forms are the two categories of endometrial cancer. Type I, which accounts for 80% of all cases, generally comprises low-grade tumors. In contrast, type II, which makes up the remaining 20% of cases, is associated with a poorer clinical prognosis [19].

A preliminary study indicated that ATR-FTIR spectroscopy could potentially differentiate between benign and malignant endometrial tissues, including their various subtypes. It seems to also have the capability to distinguish between normal tissues categorized by the menstrual cycle phase, although it was not successful in differentiating between menstrual and proliferative tissues. The most prominent variations take place in the mid- and late-secretory phases, which are distinguished by a considerable accumulation of glycogen in the former and rapid lipid alterations in the latter. Different peaks in the amide I/II regions also signal changes in structural proteins. These modifications are presumably in anticipation of embryo implantation. A key distinction between tumor and benign tissue is observed in the lipid section of the spectrum. Specifically, the lipid absorbance is most pronounced in grade 2 and 3 endometrial cancers, as well as in clear-cell tumors, adenosarcomas, and carcinosarcomas among non-endometrial cancers, potentially reflecting changes in the cell membrane, either qualitatively or quantitatively. The structural protein (amide I/II) area also shows changes, with more alterations in endometrial compared to non-endometrial tumors, especially in amide I. A significant distinction is observed between neighboring non-tumorous tissue and tumors. In endometrial cancer, the surrounding tissue resembles the proliferative phase of the endometrium more closely than in non-endometrial cancer, which is more similar to the adjacent malignancy. This might be linked to the origins of the two types of tumors. Endometrial cancer is closely connected to unopposed estrogen exposure, which induces proliferative changes in the surrounding non-cancerous tissue. These changes may be too subtle for detection via histopathology but can be identified through spectroscopy (Table 2) [20].

Endometrial carcinomas can be distinguished based on their grade, although there exists a region of overlap in the scatter plot. Clear-cell tumors exhibit a spectral signature distinct from other non-endometrial cancer types, possessing a unique molecular pattern that they share with ovarian and renal clear-cell diseases. Whether they also share spectral similarities remains uncertain [23]. Compared to control samples, shifts in amide vibrations were more significant in samples exhibiting more advanced carcinogenesis [21]. This is particularly crucial when trying to distinguish between complex atypical hyperplasia and endometrial cancer, as differential diagnosis between these two types of endometrial tissues remains challenging [24]. FTIR spectroscopy, which aids in differentiating among healthy, cancerous, and endometrial polyp tissues, is effective for detecting significant changes in pathomorphological images. However, it may not be as effective in cases where the phases of carcinogenesis are very similar. Raman spectroscopy proves to be more effective than FTIR spectroscopy in evaluating the progression of carcinogenesis in endometrial cancer. It facilitates the examination of atrophic endometrium, complex atypical hyperplasia, and the development of endometrial adenocarcinoma [21].

Analysis of the FTIR spectrum revealed a significant shift (14 cm^−1^) in the C–O stretching mode of the C–OH groups in serine, threonine, and tyrosine, from 1066 cm^−1^ in hyperplasia to 1080 cm^−1^ in carcinoma. For the amide I and amide III compounds, there was a noticeable shift toward a lower wavenumber by 3 and 4 cm^−1^, respectively. Additionally, the Raman spectra of atypical hyperplasia and cancer tissues showed a notable absence of peaks from the stretching vibrations of CO, CC, and OCH in the rings of polysaccharides and pectin, unlike in the control samples. In cancer tissues, there was a notable shift in peaks associated with tryptophan, amide I, and CH_2_ groups from lipids compared to the control samples. Moreover, PCA analysis of the Raman data demonstrated clear separation of most control samples from those obtained for cancer and atypical hyperplasia tissues. Conversely, PCA analysis of the FTIR data indicated that only the spectra from cancer tissues were similar to each other and could be differentiated from other analyzed samples. Distinction between the spectra of atypical hyperplasia and control tissues was not possible. Raman spectra of atypical hyperplasia and cancer tissues were shown to be more comparable to each other than those of the control and either atypical hyperplasia or cancer, according to Hierarchical Cluster Analysis (HCA). Regarding the FTIR data, HCA showed that cancer tissues had a greater degree of similarity than control and atypical hyperplasia samples, which did not form separate groups of similarity. Offering qualitative and quantitative insights into the vibrational modes of particular functional groups, FTIR and Raman spectroscopy are considered as complimentary techniques. Raman spectroscopy by itself is a powerful tool for distinguishing between control samples and those exhibiting malignancy and atypical hyperplasia as demonstrated by PCA and HCA. The Pearson correlation coefficient derived from the Raman data indicated a stronger correlation between the chemical compositions of atypical hyperplasia and cancer tissues compared to those of atypical hyperplasia and control tissues or cancer and normal tissues. In contrast, the Pearson correlation coefficient for the FTIR results revealed a more significant correlation between the chemical compositions of control and cancer tissues than between those with atypical hyperplasia and cancer features. This finding could be explained by the different underlying physical principles of the individual method. FTIR spectroscopy looks at changes in the dipole moment, while Raman spectroscopy is influenced by changes in bond polarity. As a result, the FTIR and Raman spectra show variations in the intensity and presence of functional groups [22].

The application of IR spectroscopy to blood and urine samples has also been studied. These approaches have demonstrated good sensitivity and specificity in detecting endometrial cancer, suggesting that blood and urine IR spectroscopy could be useful non-invasive diagnostic procedures. In a large cohort study with blood plasma samples from women diagnosed with endometrial cancer (*n* = 342), those with atypical hyperplasia (*n* = 68), and healthy individuals (*n* = 242, for a total of 652 participants) using ATR-FTIR spectroscopy combined with machine learning techniques showed that this blood-based IR spectroscopy method could identify endometrial cancer with a sensitivity of 87% and a specificity of 78%. The method showed the highest accuracy for detecting type I endometrial cancer, the most prevalent form, and atypical hyperplasia, achieving sensitivities of 91% and 100%, and specificities of 81% and 88%, respectively [25]. PCA combined with support vector machine (SVM) enabled the distinction between the two groups with a sensitivity of 100% for plasma samples (70 endometrial cancer, 15 healthy controls) on both Al foil and low-E slides. Serum samples from 60 endometrial cancer patients and 15 healthy controls were examined with IR spectroscopy with a sensitivity of 100%. For plasma samples, Al foil demonstrated a higher specificity (90%) than low-E slides (85%), but a lower specificity (70%) for serum samples. Introducing aluminum foil as a low-cost and efficient substrate may facilitate multicenter experiments and lead to its widespread use in clinics [26].

In a pilot study, urine spectroscopy effectively differentiated endometrial cancer (*n* = 109) from benign gynecological conditions (*n* = 110) with a sensitivity of 98% and a specificity of 97%. The findings remained reliable when comparing the most common endometrial cancers to controls, as well as non-endometrial cancers (including serous, clear-cell, and carcinosarcoma) to controls. Additionally, high levels of diagnostic accuracy persisted even when early stage I cancers were compared to controls, and when comparing grade 1 endometrial stage IA cancers to controls, which represent the earliest stage and grade of the disease [27]. Moreover, ATR-FTIR spectroscopy was used to examine urine samples in a study on ovarian- and endometrial cancer patients. The research identified specific biomarkers for these cancers by applying multivariate analysis and classification algorithms, leading to remarkable diagnostic accuracy: 95% sensitivity and 100% specificity for endometrial cancer, with an overall accuracy of 95%, and 100% sensitivity and 96.3% specificity for ovarian cancer, with an overall accuracy of 100% [28]. Urinary peptides and glycoproteins are potential markers for detecting endometrial cancer. In patients with endometrial cancer, increased concentrations of urinary epidermal growth factor (EGF) were measured [29,30]. The benefit of peptides found in urine is their simpler composition and greater stability compared to blood plasma proteins, making them excellent candidates for biomarker identification [31]. Elevated levels of urinary nucleic acids could indicate the unchecked cell growth typical of malignant conditions. Notably, expression levels up to 30-times higher have been detected in urinary exosomes from patients with endometrial cancer compared to controls [32]. Several potential spectral bands for classifying endometrial cancer were identified at 1485 cm^−1^ and within the range of 810–520 cm^−1^, which are attributed to, and associated with, phenyl groups. Phenylalanine is an essential amino acid, which is metabolized and excreted in urine [33]. Due to a deficiency in phenylalanine hydroxylase, there is a significant link between elevated phenylalanine levels and endometrial cancer, as it may influence the onset or advancement of the disease [34].

MIR spectroscopy analysis of dehydrated plasma or serum samples has shown the ability to distinguish endometrial cancer from benign (non-cancerous) conditions. For plasma samples analyzed within the spectral range of 1430 cm^−1^ to 900 cm^−1^, the kNN classifier demonstrated the highest performance, achieving a sensitivity, specificity, and Matthew’s correlation coefficient (MCC) of 0.865 ± 0.043, 0.865 ± 0.023, and 0.762 ± 0.034, respectively. In the same spectral range for serum, the linear discriminant analysis (LDA) classifier was the most effective, with a specificity, a sensitivity and MCC of 0.763 ± 0.048, 0.899 ± 0.023, and 0.664 ± 0.067, respectively. The support vector machine (SVM) classifier performed the best for plasma with a wider spectral range from 1800 cm^−1^ to 900 cm^−1^, achieving a specificity, a sensitivity, and MCC of 0.815 ± 0.000, 0.993 ± 0.010, and 0.815 ± 0.010, respectively. In the same extended range for serum, the quadratic discriminant analysis (QDA) was the top performer, achieving a sensitivity, specificity, and MCC of 0.852 ± 0.023, 0.700 ± 0.162, and 0.557 ± 0.012, respectively. It was noted that the performance of classifiers varied significantly between two pre-processing techniques, particularly in relation to the spectral ranges used. Classifiers tended to perform better with plasma in the wider spectral range of 1800 cm^−1^ to 900 cm^−1^ compared to the narrower range of 1430 cm^−1^ to 900 cm^−1^. Conversely, for serum, classifiers showed improved performance in the narrower range than in the wider range [18]. The reason for this difference in classifier performance between plasma and serum is the variation in their composition, specifically in terms of protein and free-DNA content. While both plasma and serum are largely composed of the same proportions (50% to 60% albumins and 40% globulins), the key distinction is the presence of fibrinogens and clotting factors in plasma, which are not found in serum [35]. The improved performance of classifiers for plasma in the 1800 cm^−1^ to 900 cm^−1^ range can be attributed to the IR signals (amide I and amide II) associated with fibrinogen [18]. This finding is corroborated by other studies [36,37], which have reported notably higher levels of fibrinogen in patients with advanced-stage endometrial cancer. The primary difference in serum between cancerous conditions and healthy controls is the elevated concentration of cell-free DNA (cfDNA) present in the former. The presence of the amide I and amide II regions diminishes the impact of the cfDNA IR signals. As a result, there is a decrease in the performance of each classifier within the 1800 cm^−1^ to 900 cm^−1^ range [18].

Several studies have demonstrated the potential of ATR-FTIR spectroscopy for the diagnosis of endometrial cancer. However, these studies frequently have small sample numbers, reducing the credibility of the results. High diagnostic accuracies, like the ones reported in pilot studies for urine spectroscopy with 98% sensitivity and 97% specificity, need to be confirmed in larger and independent cohorts to establish their reliability. Furthermore, inconsistent results may occur due to variations in spectrum analysis methodology and preprocessing approaches. Standardizing these processes is critical to ensuring reproducibility. By overcoming these limitations through comprehensive and well-designed research, the credibility of ATR-FTIR spectroscopy in identifying endometrial cancer will be increased, making it easier to integrate into ordinary clinical practice.

## 5. Ovarian Cancer

Globally, ovarian cancer ranks as the seventh most diagnosed cancer and is the eighth most common cause of cancer-related deaths in women. Compared to other gynecological malignancies, ovarian cancer has the poorest outcome and the highest mortality rate [38]. In approximately 90% of instances, ovarian cancer develops from epithelial cells. Presently, five primary types are recognized based on histopathological, immunohistochemical, and molecular genetic evaluations: high-grade serous carcinoma (accounting for 70% of cases), endometrial carcinoma (10%), clear-cell carcinoma (10%), mucinous carcinoma (3%), and low-grade serous carcinoma (comprising less than 5% of cases). The current methods, including transvaginal ultrasonography and CA-125 blood tests, lack the necessary specificity and sensitivity. The heterogeneity of ovarian tumors, with multiple subtypes exhibiting varying clinical behaviors and genetic profiles, makes universal screening protocols more complex [39,40].

Krishna et al. [14] used FTIR and Raman spectroscopy to analyze paraffin-embedded tissue samples from normal ovaries, benign ovarian tumors, and malignant ovarian neoplasms. Their findings showed that normal tissues had considerably higher protein levels and lower DNA and lipid levels than malignant tissues, which Mehrotra et al. verified [41]. Additionally, benign tissues exhibited higher protein levels and lower DNA and lipid levels than malignant tissues [14]. In a study on various types of ovarian tumors, including 35 benign ovarian tumors, 30 borderline ovarian tumors, and 106 cases of ovarian epithelial cancer encompassing serous, mucinous, clear-cell, and endometrioid types, the relative content ratios of lipid-to-protein and nucleic acid-to-carbohydrate were higher in paraffin-fixed malignant tissues, compared to those in borderline and benign tissues. Additionally, the ratio of RNA to DNA absorption peaks was lower in malignant tissues than in normal and benign tissues [42]. Analyzing eight frozen tissue samples from ovarian cancer patients with synchrotron radiation-based FTIR spectroscopy (SR-FTIR) showed elevated levels of lipids and DNA in malignant cases. Additionally, protein content was found to be higher in malignant tumors compared to borderline ovarian tumors [43]. In a study on five ovarian cancer cell lines versus a normal epithelial cell line and 12 frozen tissues from epithelial ovarian cancer patients, an increased amount of protein in ovarian cancer cell lines and an altered secondary structure of protein was observed. The ratio of 1454/1400 was found to be lower in malignant cells/tissues compared to normal cells/tissues [44]. Analyzing two cisplatin-resistant ovarian cancer cell lines (OV2008-DDP (C13) and A2780-CP) and one cisplatin-sensitive cell line (A2780), FTIR spectroscopy found that alterations in the secondary structure of proteins and a shift to higher wavenumbers associated with CH_2_ stretching vibrations were evident [45]. IR spectroscopy can differentiate normal ovaries from benign, borderline, and malignant tumors by analyzing the content and conformation of proteins, nucleic acids, and lipids. However, the findings are sometimes varied and debatable. These inconsistencies are due to different sample preparation methods (such as fresh, frozen, or paraffin-fixed samples), small sample sizes, and the intricate nature of ovarian cancer tissues in these studies [9].

When analyzing serum or plasma spectra through ATR-FTIR spectroscopy along with a proposed classification method, an accuracy rate of 96.7% was achieved in diagnosing ovarian cancer (*n* = 30) vs. 30 normal controls and an 81.7% accuracy rate in diagnosing endometrial cancer (*n* = 30) vs. 30 normal controls [46]. In another study, the diagnostic accuracy characteristics of serum and plasma in patients with ovarian cancer using the ATR-FTIR spectrum was 93.3% [47]. Lima et al. verified a 100% sensitivity and specificity in distinguishing stage I from stages II–IV ovarian cancer, along with a 94.0% sensitivity and specificity in differentiating between serous and non-serous ovarian cancers. They also achieved a 100% sensitivity and specificity in identifying ovarian cancer patients under 60 years of age versus those over 60, using plasma samples. Additionally, when analyzing spectra from serum samples of patients, the diagnostic accuracies for these categories were 91.6%, 93.0%, and 96.0%, respectively [48]. ATR-FTIR spectroscopy has been employed to evaluate urine samples from patients with ovarian cancer (*n* = 10) and subjects without (*n* = 10). The diagnostic accuracy was 100% [28].

Screening of ovarian cancer patients by FTIR spectroscopy identified key spectral features including band positions and intensities within ranges of 3000–2800 cm^−1^ (lipids), 1800–1700 cm^−1^ (phospholipids), 1700–1500 cm^−1^ (amide I and amide II groups in proteins), and 1200–900 cm^−1^ (nucleic acids), or their relative intensity ratios (Table 3) [9].

The secondary structure of proteins and specific intensity ratios, such as 3013/2958 (indicative of lipid unsaturation levels), 2925/2958 (indicative of lipid saturation levels), and 1454/1400, are notable. While there are some consistent findings across these studies, discrepancies do exist, such as in the observed differences in protein content between cancerous and normal tissues. The relatively small sample sizes are a common limitation of these studies. Thus, further research is necessary to solidify the use of FTIR spectroscopy in diagnosing ovarian cancer in larger cohorts [9].

Ovarian cancer detection by ATR-FTIR spectroscopy has shown potential. However, there are still several issues to be resolved. Results from small-sample numbers are less generalizable. Larger and more diverse study groups are necessary to fully capture the range of symptoms associated with ovarian cancer, as this disease exhibits significant variation. The use of different spectral ranges and sample preparation methods (such as fresh, frozen, or paraffin-fixed) can introduce methodological differences that may lead to inconsistencies. Developing a standardized method for sample preparation and spectrum analysis is necessary to resolve this problem. Moreover, the results could be skewed by obvious biases in the patient selection process as well as an incapacity to regulate confounding factors like the effects of radiation and chemotherapy. In-depth knowledge of the conditions of the patient and treatments is needed to address these problems.

## 6. Breast Cancer

Breast cancer is the most common cancer in women worldwide, with incidence and results varying by region, mostly affected by socioeconomic factors and access to healthcare. Early detection, generally via mammography and biopsy, is the foundation of breast cancer treatment. Nevertheless, both methods have limitations in specificity and sensitivity, making early and accurate detection challenging, particularly in dense breast tissues [49].

In a small prospective study, nine biopsies of lobular carcinoma (seven in situ and two invasive) and the adjacent healthy region of the biopsies were analyzed with IR spectroscopy. The IR spectral analysis identified three key “diagnostic spectral regions” at 3300–2850 cm^−1^, 1700–1500 cm^−1^, and 850–800 cm^−1^, corresponding to changes in membrane, collagen, and DNA structures, respectively (Table 4) [50].

The shift in the absorption band from 1161 cm^−1^ to 1172 cm^−1^ is linked to vC–O–C bonds, reflecting glycosylation processes in membrane proteins and DNA. The biomarker bands at 1172 cm^−1^ serve as diagnostic markers for cancer progression. Additionally, the shift in the absorption band from 825 cm^−1^ in B-DNA to 810 cm^−1^ in Z-DNA for grade III cancer indicates an irreversible stage of the disease. This ability to distinguish between DNA structures could enable early detection of carcinogenesis and aid in the development of novel anticancer treatments [50]. ATR-FTIR spectroscopy in conjunction with multivariate analysis has been applied to differentiate between molecular subtypes of breast cancer, based on their unique biochemical signatures. This innovative approach identifies distinctive spectral fingerprints in the ATR-FTIR spectra, which effectively segregate various breast cancer tissues from normal breast tissues. The presence of spectral biomarkers at specified wave numbers is critical to classification. The amide I and amide II bands, which are at roughly 1650 cm^−1^ and 1548 cm^−1^, respectively, are critical for determining lipid-to-protein ratios. Furthermore, phosphodiester bands at 1240 cm^−1^ and 1080 cm^−1^ play an important role in determining nucleic acid-to-lipid and phosphate-to-carbohydrate ratios. Furthermore, the range 1120 cm^−1^ to 1020 cm^−1^ is critical for calculating RNA-to-DNA and RNA-to-lipid ratios. Hormone receptor-positive tumors often show altered lipid-to-protein ratios, with pronounced changes in the amide I and amide II bands (1650 cm^−1^ and 1548 cm^−1^). These alterations imply a higher content of cellular membranes and hormone receptors, which correlates with the response of the tumor to hormonal therapy. HER2-positive tumors have distinct phosphate-to-carbohydrate ratios with spectral peaks at 1240 cm^−1^ and 1080 cm^−1^, indicating abnormalities in cellular metabolism and higher expression of the HER2 protein, which is associated with aggressive growth. Triple-negative breast cancer is a subtype which has distinct nucleic acid bands between 1120 cm^−1^ and 1020 cm^−1^, reflecting higher RNA-to-DNA ratios. These changes are indicative of rapid cellular proliferation and a lack of hormone receptors and HER2 expression, which makes triple-negative breast cancer more challenging to treat with traditional hormone therapies [56]. The Spectral-IRDx system performs absorption spectroscopy at NIR wavelengths of 850, 935, and 1060 nm. Measuring normalized detected voltages (Vdn) from deparaffinized breast biopsy tissue samples, it was possible to identify five cancerous and five normal breast tissues. Significant differences in Vdn values at 935 nm and 1060 nm were observed between the cancerous and normal tissues, indicating a potential diagnostic use. Absorption-contrast factors and volume-fraction contrasts for lipids and collagens were greater in normal tissues than in malignant ones. These findings highlight the usefulness of the Spectral-IRDx tool in delivering discrete spectral signatures that could aid in the early detection and differentiation of breast cancer tissues, supporting its potential use in clinical settings for noninvasive diagnostics [57].

A meta-analysis of data from four trials including 289 breast cancer patients found that FTIR spectroscopy on serum samples had a sensitivity of 97% and a specificity of 92%. The positive likelihood ratio (LR+) was 10.25, implying that breast cancer patients who tested positive with FTIR spectroscopy were roughly ten times more likely to have the disease than those who tested negative. The negative likelihood ratio (LR−) was 0.05, indicating a very low risk of a false-negative result among breast cancer patients. Furthermore, the area under the curve (AUC) was calculated to be 0.9729, with a Q* index of 0.9245, indicating the great sensitivity of FTIR in diagnosing breast cancer via serum analysis. The results of this study should be approached with caution, taking into account various non-threshold factors that could influence the reliability of the aggregated data [52]. The criteria for distinguishing between healthy and breast-cancer sera varied significantly across studies. Backhaus et al. [51] found that the spectrum region of 685–1250 cm^−1^ was the most efficient discriminator, but Ghimere et al. [53] and Liu et al. [58] emphasized the protein (amide I and II) region as a promising biomarker (Table 5).

Sitnikova et al. found that proteins, lipids, and carbohydrates did not have substantial discriminatory powers. Instead, they observed significant spectrum changes in nucleic acid functional groups (1250–1306 cm^−1^), highlighting the probable relevance of breast cancer-associated DNA and RNA mutations in disease progression [52]. Examining the potential of proteins as breast cancer biomarkers, Ghimere et al. [53] identified discriminatory capabilities in the amide regions (1541–1656 cm), particularly noting prominent N–H bends. Liu et al. [58] noted significant differences in the 1539–1650 cm region between healthy individuals, patients with invasive ductal carcinoma (IDC), and patients without IDC, where IR intensity was highest in the sera of healthy patients. Backhaus et al. [51] conducted a cluster analysis on three spectral regions: the CH region (2800–3100 cm^−1^), the region associated with protein, C–C, and C–H deformation (1300–1770 cm^−1^), and the region for C–O, P–O, and aromatic ring absorption (650–1200 cm^−1^). They concluded that the spectral range of 684–1250 cm^−1^ had the best prediction accuracy. Furthermore, they used artificial neural networks (ANNs) for all recorded spectra, which yielded similar results, particularly spectral changes in CH stretching vibrations (2853–2925 cm^−1^), C–O ribose, the ribose backbone, and P–O vibrations. FTIR spectroscopy of serum specimens can serve as a supplementary test to verify the presence of cancer. Since the studies in this meta-analysis did not compare benign and malignant breast tumors, or breast cancers with other types of cancer, there is insufficient evidence to confirm that FTIR spectroscopy can be effectively used for breast cancer screening [52]. Analyzing plasma samples from breast cancer patients and healthy controls, another recent study identified significant variances observed primarily at the wavenumbers 1511 cm^−1^ in the control group, and 1502 and 1515 cm^−1^ in the cancer group. These bands are indicative of protein and amide II vibrations, which vary between cancerous and non-cancerous samples. ATR-FTIR spectroscopy had an excellent performance in screening for breast cancer, with a sensitivity of 97%, a specificity of 93%, a ROC curve of 97%, and an overall prediction accuracy of 94% [54].

Furthermore, the combination of MIR spectroscopy and machine learning techniques for the rapid screening and differentiation of breast and lung cancers revealed significant spectral differences in serum samples at specific intervals (1318.59–1401.03 cm^−1^, 1492.15–1583.27 cm^−1^, and 1597.25–1721.64 cm^−1^). These discrepancies are most likely due to variances in the absorption of critical chemical bonds in protein molecules, which indicate the existence of cancer. More specifically, the absorption of important lipid and phospholipid components occurs between 1318.59 and 1515.77 cm^−1^. The spectral alterations that distinguished breast cancer from the normal population were mostly found in the ranges of 1492.15–1583.27 cm^−1^ and 1597.25–1721.64 cm^−1^. These regions primarily correlate with the absorption spectra of C=C, C=O, C=N, N–H, and O–H bonds. On a dataset of 301 training cases and 50 test cases, a study revealed that the machine learning model based on a k-nearest neighbors (KNN) algorithm exhibited the best performance, achieving 100% prediction accuracy on the test samples [55].

The use of infrared cavity ring-down spectroscopy (IR-CRDS) to identify breast cancer from breath samples has been studied. This technology, which examines exhaled volatile organic compounds (VOCs), offers a novel approach to breast cancer screening that is unaffected by breast density, a shortcoming of standard mammography. In this study, alveolar breath samples of 111 participants were collected and analyzed using IR-CRDS at four different desorption temperatures. The authors focused on the measured absorption spectra, with a special emphasis on important VOCs associated with breast cancer, which can be detected at specific wavenumbers in the IR spectrum. An SVM model demonstrated a sensitivity of 90% and a specificity of 95%, indicating a high accuracy in distinguishing between cancerous and non-cancerous samples, irrespective of breast density [59].

Several studies suggest the diagnostic potential of ATR-FTIR spectroscopy for breast cancer. However a rigorous appraisal reveals significant limits. Reproducibility and technological consistency provide important hurdles. The results of different investigations vary due to variances in methodology, sample types (e.g., serum, tissue), and spectrum analysis techniques. To ensure reproducibility, it is crucial to maintain consistent and standardized processes.

## 7. Discussion

It is clear that incorporating IR spectroscopy into everyday clinical practice has the potential to transform the diagnosis of gynecological cancers (Table 6). The non-invasive nature of IR spectroscopy, as well as its capacity to produce speedy data, have the potential to improve early detection tactics. As previously stated, critical spectral regions such as those corresponding to the amide I and II bands have demonstrated promising results in discriminating benign from malignant situations with high sensitivity and specificity. Furthermore, Ferguson et al. [60] conducted a comparative investigation of IR spectroscopy combined with machine learning approaches for the classification of malignant tissues, including those from gynecological malignancies. Their review emphasizes the need for using appropriate preprocessing techniques and machine learning algorithms to improve diagnostic accuracy. The study stresses the F1-score’s robustness as a model performance parameter, which has the potential to increase the reliability of diagnostic outcomes in gynecological oncology.

The exact wavenumbers within the IR spectrum that correlate to various biochemical markers provide a mechanism for not only identifying but also comprehending the underlying molecular dynamics of cancer growth. These changes are closely related to important pathways such as phosphoinositide 3-kinase (PI3K)/ protein kinase B (AKT)/ mechanistic target of rapamycin (mTOR) [61] and sterol regulatory element-binding proteins (SREBPs) [62], which play critical roles in ovarian cancer cell proliferation, apoptosis, and lipid synthesis. Modifications in the spectrum region of nucleic acids correspond to tumor suppressors and oncogenes such as breast cancer 1 and breast cancer 2 (BRCA1/2) and tumor protein p53 (TP53) [63]. The Warburg effect can be identified by modifications in the metabolism of glucose and the buildup of glycogen [64,65]. Changes in DNA and proteins are also associated with AKT activation and phosphatase and tensin homolog (*PTEN*) mutations [66]. In gynecological tumors, we also need to include the biochemical changes induced by therapy. Chemotherapy changes protein expression, lipid metabolism, and nucleic acid content, as seen by alterations in amide bands, lipid absorption regions, and nucleic acid peaks. Radiation therapy causes oxidative stress, DNA damage, and changes in cellular metabolism, which are reflected in DNA/RNA regions, protein structures, and lipid concentrations. Combining treatments leads to more noticeable changes in the spectrum, making research more challenging and necessitating the use of advanced computational methods. These changes occur gradually, so collecting accurate spectral data requires precise timing. To distinguish between changes caused by treatment and by disease progression, it is crucial to develop new statistical and machine learning techniques. This advancement will enhance the sensitivity of IR spectroscopy in monitoring treatment responses.

Intraoperative diagnostics could also be improved by using IR spectroscopy in real-time surgical imaging. One important strategy is to assist tissue assessment without requiring major resection by employing IR fiber optics in endoscopic or laparoscopic tools to offer real-time spectrum data. Handheld IR devices provide fast feedback on tissue composition, allowing for better tumor-margin judgments. Combining IR spectroscopy with optical coherence tomography (OCT) or fluorescence imaging yields rich structural and molecular information. Specialized IR spectroscopic scanning probes generate spectral maps for accurate tissue removal. Integration into robotic surgical equipment allows for automatic spectrum analysis, which improves accuracy. Advanced algorithms and machine learning increase real-time data processing and interpretation, resulting in much better intraoperative diagnosis and surgical results.

Portable IR-spectroscopy devices offer the potential to democratize early cancer detection by bringing this vital technology to low-resource settings, potentially improving global cancer outcomes. There is a call for further multidisciplinary research and collaboration, including large-scale clinical trials, to assess the technology in various populations. Moreover, continual technological advancements to enhance the therapeutic usefulness of spectroscopic devices is needed. IR spectroscopy might serve as the cornerstone for a new diagnostic paradigm, offering a noninvasive, cost-effective, and widely available tool for successfully diagnosing and controlling gynecological cancers. Thus, the future holds the prospect of not only increasing patient survival but also improving quality of life, demonstrating the revolutionary potential of IR spectroscopy in gynecological oncology.

Due to its high sensitivity, FTIR spectroscopy has great potential for detecting biological compounds. However, there are several limitations that hinder its widespread use in regular clinical practice. The heterogeneity introduced during sample preparation can compromise the consistency of results. Moreover, obtaining precise and clear spectra consistently can be challenging due to difficulties with spectral resolution and signal-to-noise ratio. To combat data variability, comprehensive statistical models and processes must be employed to account for all confounding variables while producing reliable and consistent results. Principal component analysis (PCA) and partial least squares (PLS) regression can reduce data dimensionality while preserving important information. Preprocessing techniques, such as smoothing, baseline correction, and normalization, can help reduce noise and variability. For data analysis, supervised learning techniques like Support Vector Machines (SVMs), random forests, and neural networks, when combined with cross-validation, ensure the resilience of the analysis. Hierarchical models are capable of handling repeated measurements while accounting for both random and fixed influences. Unsupervised learning methods, such as k-means and hierarchical clustering, can reveal patterns, while ANOVA/MANOVA can identify group differences. Bayesian approaches utilize prior information to address uncertainty, resulting in greater accuracy and dependability. To effectively analyze large-scale datasets, it is crucial to have automated analysis tools and user-friendly applications. Additionally, the limited penetration of IR light into biological tissues, reaching only a few millimeters or micrometers, presents a significant challenge when using it in deep-tissue analysis. Another drawback is the specialized skills required to utilize and analyze the data. Integrating FTIR spectroscopy into clinical operations is highly challenging because substantial adjustments are necessary compared to current diagnostic techniques. Regulatory barriers and the need for extensive validation studies to ensure the clinical relevance and reliability of FTIR-based diagnostics may also impede their adoption. Addressing these challenges is crucial for the successful translation of FTIR spectroscopy from the laboratory to clinical settings. To facilitate its routine clinical application, future research should focus on improving sample preparation protocols, data analysis techniques and software tools, reducing costs, developing comprehensive training programs, and conducting rigorous validation studies.

This review is restricted by several limitations. It is important to note that selection bias may have influenced this narrative review, as only papers deemed significant by us were included. Furthermore, excluding unpublished research, event abstracts, and papers written in languages other than English could lead to publication bias and the omission of crucial information. Another limitation is the arbitrary interpretation of the articles that were used. Although efforts were made to address this by reviewing the publications, it is important to note that the review may still contain our own personal views and biases.

To establish the clinical efficacy of IR spectroscopy, extensive population-based studies comparing it to established procedures such as Pap smears, HPV testing, and histopathology are required. The challenges include early application of IR spectroscopy, institutional norms, and negotiating regulatory and ethical issues. To prove IR spectroscopy as a viable alternative or addition, large-scale multicenter studies must evaluate its efficacy, dependability, and cost-effectiveness, while taking into account population demographics, cancer prevalence, and a variety of healthcare settings. Implementing IR spectroscopy in therapeutic settings without enough long-term efficacy data poses significant ethical challenges. Ensuring patient safety is crucial, as insufficient validation can result in incorrect diagnosis or inefficient treatments. Patients must be fully informed about the instrument’s experimental nature, including potential benefits, risks, and limits, in order to obtain informed consent. Equal access is essential for the elimination of future healthcare disparities. It is crucial to inform patients and healthcare professionals about the experimental nature of the equipment and any limitations in data. Compliance with the legislation enhances both patient safety and system reliability. Regular clinical trials are vital for establishing long-term efficacy outcomes. When evaluating new technology, clinicians should prioritize their patients’ needs. To safeguard patient autonomy, conventional diagnostic alternatives must be offered. Although needed, but not implemented in clinical practice yet, recent improvements in IR spectroscopy aim to reduce prices and increase accessibility, especially in low-resource areas. Miniaturization with micro-electro-mechanical systems (MEMSs) technology has resulted in low-cost, portable gadgets. IR detectors and quantum cascade lasers have enhanced performance and reduced costs. Integration with smartphones leverages mobile computing capacity, allowing for portable, user-friendly solutions that can be tested right away. Automated data analysis and cloud computing eliminate the need for on-site knowledge, while battery-powered spectrometers enable use in places with limited energy. Simplified user interfaces and low-cost disposable sample attachments, such as ATR crystals, reduce total expenses and maintenance. Advanced software techniques, such as artificial intelligence, improve data interpretation, making IR spectroscopy feasible and accessible in a variety of therapeutic settings. Finally, regular use of calibrated instruments with specified settings is critical. Data collecting specifications must also be defined, such as the number of scans, the velocity, and the environment. Regular use of preprocessing procedures such as baseline correction and normalization is required to ensure consistency. It is essential to utilize established statistical methods when analyzing and interpreting data. In both the planning and execution stages of clinical trials, it is important to maintain consistent sample sizes, reveal performance metrics, and select patients effectively. Adhering to the guidelines set by agencies like the FDA and EMA is crucial.

## Figures and Tables

**Table 1:** Infrared spectroscopy in cervical cancer diagnosis.

Wavenumber (cm^−1^)	Biomolecule Association	Diagnostic Significance	Ref.
1650, 1655	Amide I (Protein secondary structure, α-helical)	Indicates protein structure variations linked to cancer progression	[6]
1665	Amide I (Protein secondary structure, random coils)	Linked to changes in protein structure in cancer cells	[6]
1635	Amide I (Protein secondary structure, β-pleated)	Associated with specific structural changes in proteins	[6]
1544	Amide II (Protein linkage)	Reflects protein interaction changes in cancer	[7]
1450	Methyl/methylene bending (Proteins)	Indicates changes in lipid metabolism in cancer cells	[7]
1400	Carboxylate stretching (Proteins and Lipids)	Reflects alterations in cell metabolism linked to cancer	[7]
1305	Amide III (Protein tertiary structure)	Associated with protein conformation changes in cancer	[7]
1735	Ester carbonyl (Lipids)	Indicates lipid changes, often linked to cancerous alterations	[7]
3000–2800	CH stretching (Lipids)	Reflects significant lipid metabolism variations in cancer cells	[7]
1244, 1225	Phosphodiester bonds (Nucleic acids)	Key in identifying DNA structural variations between A-DNA and B-DNA	[8]
1080, 1150, 1055	C–O stretching (Glycogen)	Glycogen content changes indicative of cancerous transformations	[9]
970	New band emergence in cancerous cells	Indicates significant molecular changes associated with cancer	[9]

**Table 2:** ATR-FTIR spectroscopy in endometrial tissue analysis.

Wavenumber (cm^−1^)	Biomolecule Association	Diagnostic Significance	Ref.
1735	Ester carbonyl (Lipids)	Prominent in certain aggressive cancer types, reflecting cell membrane alterations	[20]
Amide I/II regions	Structural proteins	Greater variations observed in endometrial cancers, crucial for distinguishing tumor subtypes	[20]
3000–2800	CH stretching (Lipids)	Indicates lipid metabolism variations significant in cancer diagnosis	[20]
1066, 1080	C–O stretching in serine, threonine, tyrosine	Shift from hyperplasia to carcinoma shows significant biochemical changes	[21]
1485	Phenyl groups	Associated with phenylalanine metabolism changes, potential cancer biomarker	[22]
810–520	Broad band for phenyl groups	Indicative of metabolic alterations in endometrial cancer	[22]

**Table 3:** ATR-FTIR spectroscopy in ovarian cancer analysis.

Wavenumber (cm^−1^)	Biomolecule Association	Diagnostic Significance	Ref.
3000–2800	C-H stretching (Lipids)	Indicates lipid content; higher levels in normal tissues compared to malignant tissues	[9]
1800–1700	Phospholipids	Used to assess tumor lipid profiles, varying between benign and malignant tissues	[9]
1700–1500	Amide I and II (Proteins)	Key protein bands differ significantly between benign, borderline, and malignant tissues	[9]
1200–900	Nucleic acids	Variations indicate changes in DNA/RNA structures between normal and cancerous tissues	[9]
2925/2958	Lipid saturation levels	Helps differentiate levels of lipid saturation between normal and malignant cells	[9]
3013/2958	Lipid unsaturation levels	Indicates unsaturation levels, differs in cancerous versus normal ovarian tissues	[9]
1454/1400	Protein and lipid ratios	Lower ratios in malignant cells/tissues indicate changes in protein–lipid interactions	[44]

**Table 4:** ATR-FTIR spectroscopy in breast cancer analysis.

Wavenumber (cm^−1^)	Biomolecule Association	Diagnostic Significance	Ref.
685–1250	General spectral region	Identified as the most effective discriminator between healthy and breast cancer sera	[51]
1250–1306	Nucleic acids	Significant spectral changes associated with DNA and RNA alterations in breast cancer	[52]
1541–1656	Amide I and II (Proteins)	Discriminatory capabilities in the amide regions, notable N-H bends in breast cancer	[53]
2800–3100	CH stretching (Lipids)	CH region analysis used to cluster spectral data, showing differences in lipid content	[51]
1300–1770	Protein, C–C, and C–H deformation	Region used to analyze protein content differences in breast cancer versus healthy tissues	[51]
650–1200	C–O, P–O, and aromatic ring absorption	Effective for predicting breast cancer presence through spectral analysis	[51]
1511, 1502, 1515	Amide II (Proteins)	Significant variances in amide II vibrations between cancerous and non-cancerous samples	[54]
1318.59–1401.03	Lipids and phospholipids	Identified significant spectral differences in the absorption of key chemical bonds	[55]
1492.15–1583.27	C=C, C=O, C=N	Regions showing spectral differences in protein molecules, indicative of cancer presence	[55]
1597.25–1721.64	N–H, O–H	Key regions in distinguishing breast cancer from normal samples in spectral analysis	[55]
850–800	DNA structures	Changes in membrane and DNA structures detected, useful for early cancer detection	[50]
1172	vC–O–C bonds	Reflects glycosylation processes in membrane proteins and DNA, marker for cancer progression	[50]
810	Z-DNA	Shift in absorption band indicative of an irreversible stage in grade III cancer	[50]

**Table 5:** ATR-FTIR spectroscopy in breast cancer analysis.

Wavenumber (cm^−1^)	Biomolecule Association	Diagnostic Significance	Ref.
850–800	DNA structures	Changes in membrane and DNA structures detected, useful for early cancer detection	[50]
1172	vC–O–C bonds	Reflects glycosylation processes in membrane proteins and DNA, marker for cancer progression	[50]
810	Z-DNA	Shift in absorption band indicative of an irreversible stage in grade III cancer	[50]
685–1250	General spectral region	Identified as the most effective discriminator between healthy and breast-cancer sera	[51]
2800–3100	CH stretching (Lipids)	CH region analysis used to cluster spectral data, showing differences in lipid content	[51]
1300–1770	Protein, C–C, and C–H deformation	Region used to analyze protein content differences in breast-cancer versus healthy tissues	[51]
650–1200	C–O, P–O, and aromatic ring absorption	Effective for predicting breast cancer presence through spectral analysis	[51]
1250-1306	Nucleic acids	Significant spectral changes associated with DNA and RNA alterations in breast cancer	[52]
1541–1656	Amide I and II (Proteins)	Discriminatory capabilities in the amide regions, notable N-H bends in breast cancer	[53]
1511, 1502, 1515	Amide II (Proteins)	Significant variances in amide II vibrations between cancerous and non-cancerous samples	[54]
1318.59–1401.03	Lipids and phospholipids	Identified significant spectral differences in the absorption of key chemical bonds	[55]
1492.15–1583.27	C=C, C=O, C=N	Regions showing spectral differences in protein molecules, indicative of cancer presence	[55]
1597.25–1721.64	N–H, O–H	Key regions in distinguishing breast-cancer from normal samples in spectral analysis	[55]
1597.25–1721.64	N–H, O–H	Key regions in distinguishing breast-cancer from normal samples in spectral analysis	[55]

**Table 6:** Overview of the main results of the studies investigating vibrational spectroscopy in cervical, endometrial, ovarian and breast cancer.

Disease	Study Population	Year	Major Findings	Ref.
Cervical Cancer	Exfoliated cervical cells from 156 females, of whom 136 were healthy, 12 had cervical cancer, and 8 had dysplasia.	1991	In malignant samples: (i) significant changes in the intensity of the glycogen bands at 1025 cm^−1^ and 1047 cm^−1^, the bands at 1082 cm^−1^ and 1244 cm^−1^, the C–O stretching band at 1155 cm^−1^, and the band at 1303 cm^−1^; (ii) significant shifts of the peaks normally appearing at 1082 cm^−1^, 1155 cm^−1^, and 1244 cm^−1^; and (iii) an additional band at 970 cm^−1^.	[11]
	Cervical samples from 436 females.	1997	The sensitivity of FTIR for cervical cancer detection was 79%, specificity was 77%, positive predictive value was 15%, and negative predictive value was 98.6%.	[8]
	Exfoliated cervical cells from 272 patients.	1996	The PCA score plot indicated broad clustering of the visually categorized spectra.	[13]
	Five patients with HSIL and five patients with LSIL.	2004	The amide I and II area (1740–1470 cm^−1^) were crucial for identifying anatomical and histological characteristics.	[6]
	A total of 35 cervical tissues, including 17 squamous cell carcinoma of cervical samples, 5 adenocarcinoma of cervical samples, and 13 normal cervical samples.	2006	The three different types of tissues showed significant variations in relative absorbance ratios at 1080, 1238, 1314, 1339, 1397, 1454, 1541, 1647, 2854, 2873, 2926, and 2958 cm^−1^.	[9]
	Seventeen patient samples: five normal, five LSIL encompassing HPV, two normal with history of abnormality, three normal hrHPV−, one normal hrHPV+ and one LSIL hrHPV+.	2010	SCP differentiated cytopathological diagnoses between 12 distinct cervical samples with good specificity and sensitivity. SCP also found two samples with anomalous spectral changes. They had a benign cytopathological diagnosis but a history of abnormal cervical cytology. The spectrum alterations found in the morphologically normal cells are most likely the result of an HPV infection. SCP correctly discriminated these samples according to their HPV status.	[12]
Endometrial cancer	A total of 126 blood samples were collected from 31 endometrial plasma cancer patients, 32 endometrial plasma control, 30 endometrial serum cancer patients, and 33 endometrial serum control patients, prior to surgery.	2021	KNN of plasma samples (with spectral data spanning from 1430 cm^−1^ to 900 cm^−1^) achieved a sensitivity, specificity, and MCC of 0.865 ± 0.043, 0.865 ± 0.023, and 0.762 ± 0.034. LDA of serum samples (in the same wavenumber range) showed a sensitivity, specificity, and MCC of 0.899 ± 0.023, 0.763 ± 0.048, and 0.664 ± 0.067. SVM on plasma (with spectral data ranging from 1800 cm^−1^ to 900 cm^−1^) resulted in a sensitivity, specificity, and MCC of 0.993 ± 0.010, 0.815 ± 0.000, and 0.815 ± 0.010. QDA of serum had the highest sensitivity, specificity, and MCC in the same wavenumber range, with values of 0.852 ± 0.023, 0.700 ± 0.162, and 0.557 ± 0.012.	[18]
	Tissue was taken from 76 women undergoing a hysterectomy, of whom 36 had endometrial cancer.	2011	The score plot of the LDA showed significant overlap between the three groups. However, drawing a line perpendicular to LD1, at the point of origin, allowed for around 80% separation between benign and malignant spectra.	[20]
	Five groups: control (17 tissues); atrophic endometrium (12 tissues); complex atypical hyperplasia (8 tissues); endometrial polyp (6 tissues); endometrioid adenocarcinoma (16 tissues).	2021	Raman spectroscopy is more effective than FTIR spectroscopy in assessing the development of carcinogenesis in endometrial cancer.	[21]
	Tissue samples were collected from 45 patients: 16 of them had endometrial cancer, 12 had atypical hyperplasia, and 17 were normal.	2020	PCA analysis of the FTIR data revealed that only the spectra of cancer tissues were similar to one another and could be separated from the other analyzed samples. It was impossible to identify the spectra of atypical hyperplasia from normal tissues. HCA analysis of the FTIR data revealed only a resemblance between practically all cancer tissues. However, control and atypical hyperplasia samples did not form comparable groups.	[22]
	Blood plasma and serum samples from women with endometrial cancer (*n* = 70) and healthy controls (*n* = 15).	2020	PCA and SVM models of both serum and plasma samples showed a sensitivity of 100%.	[25]
	Blood plasma samples of women with endometrial cancer (*n* = 342), its precursor lesion atypical hyperplasia (*n* = 68), and healthy controls (*n* = 242, total *n* = 652).	2020	Blood-based IR could diagnose type I endometrial cancer with 87% sensitivity and 78% specificity. It was most accurate for type I endometrial cancer and atypical hyperplasia, with sensitivity of 91% and 100%, and specificity of 81% and 88%, respectively.	[26]
	Urinary samples of patients with endometrial cancer (*n* = 109) and benign gynecological disorders (*n* = 110).	2022	Urine spectroscopy discriminated endometrial cancer from benign gynecological lesions with 98% sensitivity and 97% specificity.	[27]
	Urine samples were collected from women with endometrial (*n* = 10) and ovarian cancer (*n* = 10), as well as healthy persons (*n* = 10).	2018	Multivariate data analysis resulted in high levels of accuracy for both endometrial (sensitivity: 95%, specificity: 100%, accuracy: 95%) and ovarian cancer (sensitivity: 100%, specificity: 96.3%, accuracy: 100%).	[28]
	Urinary samples of patients with endometrial cancer (*n* = 109) and benign gynecological disorders (*n* = 110).	2022	Urine spectroscopy discriminated endometrial cancer from benign gynecological lesions with 98% sensitivity and 97% specificity.	[27]
Ovarian cancer	A total of 24 ovarian tissue specimens comprising 8 normal, 10 benign and 6 malignant tissues were recruited.	2007	Cluster analysis of second-derivative FTIR spectra in the combined spectral bands of 1540–1680 and 1720–1780 cm^−1^ revealed two distinct clusters, corresponding to malignant and normal + benign tissues. The cluster corresponding to normal + benign tissues generated nonoverlapping subclusters for normal and benign tissues with lower heterogeneity levels.	[14]
	Tissue samples of 12 cases of ovarian cancer.	2010	There were significant spectral discrepancies between normal and malignant ovarian tissues. Changes in frequency and intensity were detected in the spectrum area of protein, nucleic acid, and lipid vibrational modes.	[41]
	There were 35 histologically benign ovarian samples, 30 with borderline ovarian tumors, and 106 with epithelial carcinoma included.	2016	PCA revealed clear segregation between benign, borderline, and malignant tumors, as well as segregation between different histological tumor subtypes.	[42]
	Eight samples representing various forms of ovarian tumors were examined.	2018	Changes in chemical composition of phosphate groups and lipids might be able to differentiate between borderline and malignant ovarian tumors. In instances of cancer, there was an elevated concentration of lipids and other groups, including DNA. A rise in protein content was noted in the case of initial tumors.	[43]
	Normal and cancerous tissue samples from 12 ovarian cancer patients.	2018	Specific alterations included a reduction in the quantity of lipids and nucleic acids in malignant cells. Certain cancer cells also showed changes in the content and shape of proteins. In normal cells and tissues, the ban-intensity ratio of 1454/1400 cm^−1^ was greater, but in cancerous cells and tissues, it was lower.	[44]
	FTIR spectra of types of A2780, A2780-CP and C13 cell lines.	2012	The spectrum of the cisplatin resistance pattern was typified by a shift toward the high wavenumbers of CH_2_ stretching vibration and a conformational change in the secondary structure of proteins. Using two PCs, PCA accurately identified 96% of all spectra, providing a satisfactory separation for depicting the range of spectra from resistant and sensitive cell lines.	[45]
	Thirty ovarian cancer patients, thirty endometrial cancer cases, and thirty non-cancer controls provided plasma and serum samples.	2013	While endometrial cancer was recognized with a relatively good accuracy (up to 81.7%), classification findings for ovarian cancer were exceptional (up to 96.7%).	[46]
	Blood samples taken from 30 patients with ovarian cancer and 30 healthy controls.	2014	SVM of blood plasma’s Raman spectra were classified with 74% diagnostic accuracy. The same classifier demonstrated 93.3% accuracy for the blood plasma’s IR spectra.	[47]
	A total of 30 plasma samples and 30 serum samples were taken from a total of 30 individuals with different stages of ovarian cancer.	2015	A GA-LDA model with 33 wavenumbers was used to obtain 100% sensitivity and specificity for differentiating between stage I and stages II–IV. Using 29 wavenumbers via GA-LDA, the sensitivity and specificity scores for the serous vs. non-serous categories were up to 94%. Using 42 wavenumbers via GA-LDA, the sensitivity and specificity provided 100% accuracy for the ≤60 years and >60 years categories. Using several wavenumbers, the sensitivity and specificity findings for blood samples showed reasonably good accuracy (up to 91.6% for stage I vs. stages II–IV, 93.0% for serous vs. non-serous, and 96.0% for ≤60 years vs. >60 years).	[48]
Breast cancer	A total of 196 patients with breast cancer.	2010	Unsupervised cluster analysis was able to obtain 98% sensitivity and 95% specificity. ANN revealed a 92% sensitivity and a 100% specificity.	[51]
	A total of 20 individuals were included: 10 healthy controls and 10 patients with breast cancer.	2020	The ratio of the α-helix to the β-pleated sheet in proteins had 90% sensitivity and specificity. Similarly, the amide II and III ratio (I1556/I1295) demonstrated 100% and 80% sensitivity and specificity, respectively.	[53]
	Blood plasma of breast cancer patients and healthy controls.	2023	ATR-FTIR spectroscopy achieved 97% sensitivity, 93% specificity, 97% ROC curve, and 94% prediction accuracy in differentiating between breast cancer patients and healthy controls.	[54]
	Serum samples of 98 breast cancer patients and 158 healthy controls.	2023	With 100% prediction accuracy on test set samples, the prediction model trained using the KNN architecture exhibits the highest performance.	[55]
	Tissue sections were obtained from seven people with breast fibroadenoma, seven patients with breast cancer, and seven patients with normal breast tissue.	2021	2D-PCA-LDA showed clear clustering of both groups.	[56]
	Ten samples of deparaffinized breast biopsy tissue were used; five samples were cancerous and five were normal.	2020	There was a statistically significant difference in normalized detected voltage between cancer and normal tissues at 935 and 1060 nm, with *p*-values of 0.0038 and 0.0022, respectively. Furthermore, the volume fraction contrast (N/C) of lipid (∼1.28) indicates that normal tissue has greater lipid levels than malignant tissue.	[57]
	Serum samples from 41 non-IDC patients, 74 IDC patients, and 114 healthy people.	2020	With an accuracy of 95.7%, sensitivity of 91.7%, and specificity of 100%, the polynomial kernel produced the best results.	[58]
	Alveolar breath samples of 111 people in total (71 positive and 40 control) were included.	2023	Using IR-CRDS to classify alveolar breath could be a potential method for breast cancer screening that is independent of breast density.	[59]

Abbreviations. HSIL: high-grade squamous intraepithelial lesion; LSIL: low-grade squamous intraepithelial lesion; HPV: human papillomavirus; hrHPV: high-risk human papillomavirus; SCP: spectral cytopathology; DNA: deoxyribonucleic acid; PCA: principal component analysis; LDA: linear discriminant analysis; LD: linear discriminant function; FTIR: Fourier-transform infrared spectroscopy; HCA: hierarchial cluster analysis; SVM: support vector machines; IR: infrared; KNN: K-nearest neighbour; MCC: Matthews correlation coefficient; QDA: quadratic discriminant analysis; PC: principal components; ATR-FTIR: attenuated total reflectance–Fourier-transform infrared; GA-LDA: genetic algorithm-linear discriminant analysis; ANN: artificial neural networks; ROC: receiver operating curve; IR-CRDS: infrared cavity ring-down spectroscopy.
